# Health care use, drug treatment and comorbidity in patients with schizophrenia or non-affective psychosis in Sweden: a cross-sectional study

**DOI:** 10.1186/s12888-017-1582-x

**Published:** 2017-12-29

**Authors:** Erica M. Brostedt, Mussie Msghina, Marie Persson, Björn Wettermark

**Affiliations:** 1 Department of Healthcare Development, Public Health Care Services, Stockholm County Council, Box 6909, 102 39 Stockholm, Sweden; 2 Department of Medicine, Clinical Epidemiology Unit T2, Centre for Pharmacoepidemiology, Karolinska University Hospital, Karolinska Institutet, Stockholm, 17176 Sweden; 3 Department of Clinical Neuroscience, Centre for Psychiatric Research, Karolinska Universitetssjukhuset, Huddinge, Karolinska Institutet, Stockholm, 141 86 Sweden; 4 Pharmaceutical Unit, Public Health Care Services, Stockholm County Council, Box 17533, Stockholm, 11891 Sweden

**Keywords:** Schizophrenia, Delivery of health care, Comorbidity, Antipsychotic agents, Delayed-action preparations

## Abstract

**Background:**

This study investigated the prevalence of schizophrenia (ICD-10 F 20) and of other non-affective psychosis (NAP, ICD-10 F 21 - F 29) in Sweden. It further assessed health care use, comorbidity and medication for these patient groups.

Most studies either have a study population of patients with strictly defined schizophrenia or a psychosis population of which strict schizophrenia cases form a smaller set. The present study permits comparison of the two mutually exclusive patient groups using data at the individual level in the diagnosis of non-affective psychosis, use of health care, medical treatment and comorbidity by diagnosis or medical treatment.

**Methods:**

In 2012, data were extracted from a regional registry containing patient-level data on consultations, hospitalisations, diagnoses and dispensed drugs for the total population in the region of Stockholm (2.1 million inhabitants). The size of the total psychosis population was 18,769, of which 7284 had a diagnosis of schizophrenia. Crude prevalence rates and risk rates with 95% confidence intervals were calculated.

**Results:**

In 2012, the prevalence of schizophrenia and NAP was 3.5/1000 and 5.5/1000, respectively. Schizophrenia was most common among patients aged 50–59 years and NAP most common among patients aged 40–49 years.

Schizophrenia patients used psychiatric health care more often than the NAP patients but less overall inpatient care (78.6 vs. 60.0%).

The most prevalent comorbidities were substance abuse/dependence (7.9% in the schizophrenia group vs. 11.7% in the NAP group), hypertension (7.9 vs. 9.7%) and diabetes (6.9 vs. 4.8%).

The parenteral form of long-acting injectable antipsychotics was more often dispensed to patients with schizophrenia (10 vs. 2%).

**Conclusions:**

This study, analysing all diagnoses recorded in a large health region, confirmed prevalence rates found in previous studies. Schizophrenia patients use more psychiatric and less overall inpatient health care than NAP patients. Differences between the two patient groups in comorbidity and drug treatment were found. The registered rates of a substance abuse/dependence diagnosis were the most common comorbidity observed among the patients investigated. The observed differences between the schizophrenia and the NAP patients in health care consumption, comorbidity and drug treatment are relevant and warrant further studies.

## Background

Schizophrenia is a debilitating, often chronic, mental disorder that imposes a huge burden on the individual and society. Aetiological factors are related to the environment, behaviour, genetic vulnerability of the individual and perhaps an underlying common factor. According to the Global Burden of Disease (2012) study, schizophrenia accounts for 7.4% of the total amount of DALYs (disability-adjusted life years) worldwide. Furthermore, schizophrenia contributes with 7.1% of the total YLLs (years of life lost to premature mortality) of mental and substance use disorders [1]. For most patients, the disease is chronic. Studies on recovery have shown rates to vary between 4 and 13.5% [2, 3]. However, in one study complete remission was about 30% [4]. Poor disease awareness is common in the schizophrenia patient population, ranging between 27 and 57%, depending on the study [5]. By studying data on psychiatric symptoms from the social security and primary care registries on the Swedish population, an estimated hidden proportion of 2–3% of patients with psychotic symptoms had never been in contact with psychiatric care [6, 7].

For a society to estimate enough resources for mental health care needs, it is important to investigate the extent to which the actual need for mental health services is met. There is ample evidence suggesting that psychiatric care is under used. A random selection of participants in the Stockholm-based PART study was interviewed for this purpose [8]. In over 40% of the population a need for psychiatric care, which was possible to meet by the healthcare system, was identified. The proportion of those who were aware of the need for care and actually had their needs met by established treatment was 5%. Factors associated with having the need for care met were being female, higher education and good social support. In another community survey the need for care of severely mentally ill patients was investigated. A sex difference was again observed: men had problems with functional disability and women had a greater need of information on health, security and physical health [9].

Studies on medical treatment of the mentally ill usually include either a study population of patients with strictly defined schizophrenia (ICD-10 F 20) or an overlapping psychosis population in which schizophrenia cases are a subset (ICD-10 F 20 - F 29). There are effective medicines for the treatment of psychoses, but it is important to acknowledge the ongoing debate about the most appropriate dosage, choice of substance (or combination thereof) and means of treatment (daily per oral or parenteral) to achieve the most efficacious effect. As has been demonstrated in the Clinical Antipsychotic Trials of Intervention Effectiveness (CATIE) study, the response to treatment is highly variable and implies a need for more individualised treatment for optimal results [10]. Co-medication with similar drugs is common but details as to the extent of co-medication and the combinations being used are limited [11, 12]. In a Swedish national study on all patients dispensed antipsychotics (*n* = 132,000) 75% of the population were given only one antipsychotic drug [13]. However, there were over 665 unique combinations of antipsychotics dispensed in the remaining population. The most prevalent antipsychotic drug in the combinations was levomepromazine.

The low rate of patient adherence and persistence constitutes another problem related to schizophrenia treatment. In a review of the literature on non-adherence to treatment in schizophrenic patients Lacro et al. calculated a mean rate of 41% for non-adherence in the 10 studies they reviewed [14].

Comorbidity with somatic and other psychiatric disorders is not uncommon [15, 16]. The life-time risk of substance abuse in patients with schizophrenia has been estimated to be from 20 to 70% [15]. In the Epidemiologic Catchment Area (ECA) study, the prevalence of drug abuse was 47% in the schizophrenic population compared to 13% in the general population [17].

In a Finnish, general population study obesity and type 2 diabetes were more prevalent in schizophrenic patients than in the general population [18]. The results from the baseline study of the untreated schizophrenia patient population in the CATIE study showed a prevalence rate of 62% for hypertension and 30% for diabetes [19]. Crump et al. investigated somatic comorbidities and mortality in Swedish schizophrenic patients and found a shorter life span for that population. Men died ~15 years earlier and women ~12 years earlier because of somatic comorbidity. The main causes of death were cancer and ischaemic heart disease [20]. Whether the disease itself is a risk factor for some of the medical comorbidities prevalent in patients with schizophrenia or if these are the result of antipsychotic treatment remains unresolved [21]. One way to address this issue is to compare medical comorbidities in patients with schizophrenia with patients with other non-affective psychoses who also are exposed to antipsychotic medications, as we have done in this study.

The medical claims data in this study permit comparisons between the schizophrenic group and the NAP group using data at the individual level in the diagnosis of psychosis, health care use, medical treatment and comorbidity by diagnosis or medical treatment. The pharmacological treatment of schizophrenia and other non-affective psychoses is mainly based on antipsychotic medications. However, the duration of treatment may vary depending on the chronicity of the disorder, with schizophrenia and related disorders often requiring life-long treatment, whereas non-chronic psychotic disorders may require treatment only during periods of symptom manifestation.

### Aims of the study.

The study aimed to investigate the similarities and differences in patterns of health care use, medication patterns and the prevalence of psychiatric and somatic comorbidities between two mutually exclusive psychosis groups: individuals who received a diagnosis of schizophrenia (ICD-10 F 20) and individuals with a diagnosis of NAP (ICD-10 F 21 - F 29).

## Methods

### Study design

This cross-sectional study included all individuals alive on 1 Jan 2012 and living in Stockholm County. The county contains over a fifth (2.1 million individuals) of Sweden’s population and includes the capital city of Stockholm, several suburban areas and communities, large rural areas and a sparsely populated archipelago.

The healthcare system in Sweden is financed primarily through taxes levied by county councils and municipalities. Apart from the very few un-subsidised private clinics, all data on hospitalisations, outpatient visits in primary as well as specialist care and dispensed prescriptions are recorded, collected and stored in, the Stockholm County regional data warehouse (VAL). Thus, VAL facilitates epidemiological research in a large, unselected population cohort [22]. The present study was approved by the regional ethical research board in Stockholm, Sweden (2014/1307–31).

### Subjects

We identified all patients with a registered diagnosis of non-affective psychosis (ICD-10 F 20 - F 29) during hospitalisation or outpatient consultation in primary or specialist care between 1 Jan 2000 and 31 Dec 2012. Patients alive for at least one day and living in Stockholm County during 2012 who had either a recorded contact (for any reason) with any health care provider in the region or a dispensed antipsychotic medication during 2012 were included.

Within the time frame (i.e. 2000–2012), a registered diagnosis of schizophrenia (ICD-10 F 20) qualified the individual as a case of schizophrenia. As a case of NAP, anyone with a registered ICD-10 code of F 21 - F 29 within the same time frame qualified. These two diagnosis groups were considered mutually exclusive in the sense that if a patient had ever (i.e. during 2000–2012) had a diagnosis of schizophrenia recorded, the patient qualified for the schizophrenia population only.

### Comorbidity and concomitant drug treatment

Schizophrenia patients are known to have a 10–20-year shorter life expectancy compared with a healthy population. In the present study we focused on somatic co-morbidities known to be risk factors for increased mortality: obesity (E65–66), hypertension (I10-I15), diabetes type 2 (E11) and substance abuse/dependence (F10–19). Self-harm (X60–84) was included as an indicator of suicidal and para-suicidal behaviour. The selected comorbidities for all patients were analysed by determining the 1-year and 12-year prevalence of a diagnosis recorded according to the ICD-10 classification system.

Pharmacological treatments in the study population in 2012 as defined by the Anatomical Therapeutic Chemical (ATC) classification system were analgesics (i.e. opioids only, N02A), anxiolytics (N05B), sedatives (N05C), antidepressants (N06A), anti-addictives (N07B), anti-alcohol dependence (N07BB), anti-opioid dependence (N07 BC), antidiabetics (A10), antithrombotics (B01A), anti-hypertensives (C03A-C03E, C07-C09) and antipsychotic medications (N05A, excluding N05AN01 and N05AA02, i.e. lithium and levomepromazine).

### Statistical analysis

Crude prevalence rates (1 year, 2012 and 12 years, 2000–2012) for all non-affective psychosis diagnoses, comorbidity with other psychiatric and somatic disorders and dispensed antipsychotics and other medications were calculated in absolute terms and as a risk ratio (RR) with 95% confidence intervals (CIs) for both diagnostic groups. Data on health care use were presented as medians and differences between the two patient groups were assessed by Wilcoxon rank sum test/χ^2−^statistics. No adjustments due to differences in age or sex in the study population were undertaken.

## Results

### Prevalence by age and sex

Of the study population containing 18,769 patients (57% men) with non-affective psychosis, 39% had a diagnosis of schizophrenia, which translates to a 1-year prevalence for schizophrenia in the Stockholm County of 3.5/1000 inhabitants. The 1-year prevalence for the NAP patient group was estimated to be 5.5/1000 inhabitants, of which 49% were men.

The difference in median age between the populations was 5 years: median age for the schizophrenia patients was 53 years and 48 years for the NAP patients (Table 1).

**Table 1:** Population characteristics and comorbidities in schizophrenia and NAP patients in Stockholm, Sweden in 2012

	Study population			
	Schizophrenia	NAP		
N	7284	11,485		
% males	57	49		
Age (mean, SD)	52.7 (14.4)	49.5 (18.5)		
Age (median)	53 (43–62)	48 (35–62)		
1-year prevalence (%, 2012)			RR	95% CI
Diabetes^a^	6.9	4.8	1.45	1.28–1.63
Hypertension^a^	7.9	9.7	0.82	0.74–0.90
Obesity	2.2	2.2	0.97	0.8–1.18
Substance abuse^a^	7.9	11.7	0.63	0.61–0.74
Self-harm^a^	0.6	0.9	0.65	0.45–0.93
12-year prevalence (%, 2000–2012)				
Diabetes^a^	12.5	7.9	1.58	1.44–1.72
Hypertension^a^	17.0	19.3	0.88	0.83–0.94
Obesity^a^	9.9	8,0	1.23	1.12–1.35
Substance abuse^a^	24.8	28.4	0.87	0.83–0.92
Self-harm^a^	3.5	4.3	0.82	0.70–0.95

Note: If ever (2000–2012) diagnosed with schizophrenia, then considered part of the schizophrenia group only, irrespective of NAP ever diagnosed

^a^statistically significant difference in RR between the two diagnostic groups (95% CI for RR)

**Fig. 1:**
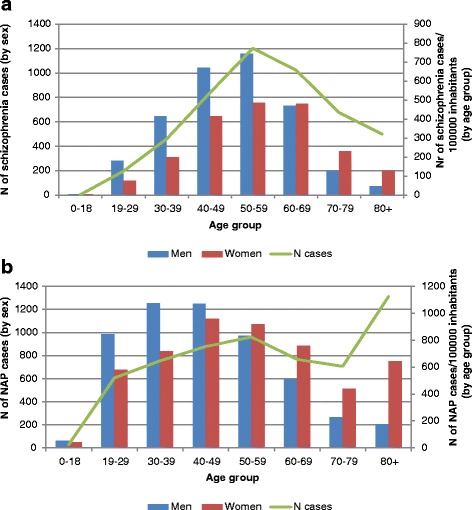
**a** Distribution of schizophrenia cases registered from 2000 to 2012 as a function of age group and sex. **b** Distribution of NAP cases registered from 2000 to 2012 as a function of age group and sex

The age group with the highest prevalence was the 50–59-year olds for the schizophrenia group and the 40–49-year olds for the NAP group. Male cases of schizophrenia dominated in the younger age groups and female cases dominated in the older age groups (Fig. 1a and b). The difference in prevalence rates between the two populations was statistically significant for all age groups, but of borderline significance in the age groups of 40–49-year olds and 70–79-year olds. The high prevalence among the oldest age group (80+) observed in the NAP population was not seen in the schizophrenia population (1124/100000 inhabitants vs. 321/100000 inhabitants).

### Health care use

As shown in Table 2, the schizophrenia patients, in comparison with the NAP population, used psychiatric care more often. Moreover, the schizophrenia patients used outpatient care more often, i.e. they had more outpatient visits, somatic as well as psychiatric, than the NAP patients. There were statistically significant differences between the patient groups for all levels of health care, except for those who only had inpatient care. There were small but significant differences in the number of visits or bed days.

**Table 2:** Proportion of patients (%) and medians for health care use during 2012 by patient group

	Schizophrenia (%)	NAP (%)	RR	95% CI
Inpatient care^a^	25.01	29.34	0.85	0.82–0.90
Outpatient care^a^	84.10	76.69	1.08	1.08–1.11
Primary care	63.29	66.49	0.95	0.78–1.17
Psychiatric care^a^	78.64	60.04	1.31	1.28–1.33
No Psychiatric care^a^	21.36	39.96	0.54	0.51–0.56
	Schizophrenia (medians, range)	NAP (medians, range)	Wilcoxon rank sum test/χ^2^ statistics	*P*-value
Primary care consultations ^b^	2 (0–1142)	2 (0–1023)	18.51	<0.0001
Outpatient consultations ^b^	23 (0–1303)	14 (0–2335)	319.94	<0.0001
Outpatient psychiatric consultations^b^	13 (0–1123)	3 (0–226)	879.01	<0.0001
Inpatient admissions ^b^	0 (0–93)	0 (0–45)	46.75	<0.0001
Inpatient psychiatric admissions^b^	0 (0–63)	0 (0–39)	13.72	0.0002
Inpatient care, days^b^	0 (0–364)	13 (1–336)	25.77	<0.0001
Inpatient psychiatric care, days^b^	0 (0–364)	0 (0–337)	5.82	0.02

Note: The categories ‘Inpatient care’ and ‘Outpatient care’ include somatic and psychiatric consultations. ‘Psychiatric care’ includes inpatient and outpatient psychiatric care. Psychiatric care given at the primary level of care is not included

^a^Statistically significant difference in RR between the two diagnostic groups (95% CI for RR)

^b^Statistically significant difference between the two diagnostic groups by Wilcoxon rank sum test/χ^2^ statistics

In the study population there were 7670 patients (41%, 55% men) who had health care consultations in 2012 but were not dispensed any antipsychotic prescriptions, apart from potential medication administered in a hospital setting. Of those 7670 patients, 21% had a diagnosis of schizophrenia.

### Treatment with antipsychotics

As shown in Table 3, there was a plethora of antipsychotic medications prescribed and dispensed to the psychotic patients. Even the most commonly dispensed substance was not dispensed to more than 21.4% of the schizophrenia patients and 16.4% of the NAP patients.

**Table 3:** Antipsychotic treatment, route of distribution and substance by proportion of patients with dispensed prescription in 2012

	Schizophrenia (%)	NAP (%)	RR	95% CI	
Antipsychotic medication	(*N* = 7284)	(*N* = 11,485)			
Per oral^a^	55.5	41.9	1.32	1.29–1.36	
Long-term injectables^a^	10.3	2.05	5.01	4.35–5.79	
Per oral and long-term injectables^a^	12.5	2.9	4.26	3.78–4.81	
No antipsychotic medication^a^	21.6	53.1	0.41	0.39–0.43	
Substance	Schizophrenia (%)	NAP (%)	RR	95% CI	
Olanzapine^a^	21.4	16.4	1.33	1.25–1.41	
Zuclopenthixol^a^	14.1	3.3	4.24	3.78–4.75	
Risperidone^a^	12.9	9.9	1.30	1.20–1.41	
Perphenazine^a^	12.8	4.7	2.72	2.45–3.01	
Haloperidol^a^	12.6	6.4	1.95	1.78–2.14	
Clozapine^a^	11.6	1.2	9.63	8.06–11.50	
Aripiprazole^a^	9.2	6.0	1.54	1.39–1.70	
Quetiapine^a^	7.1	8.2	0.86	0.78–0.95	
Flupentixol^a^	3.9	2.7	1.42	1.21–1.66	
Paliperidone^a^	3.8	1.3	2.92	2.40–3.56	
Ziprasidone	1.4	1.0	1.30	1.00–1.69	
Chlorprothixene	0.8	0.06	1.33	0.93–1.91	
Fluphenazine^a^	0.3	0.05	6.31	2.58–15.42	
Melperone	0.2	0.1	1.58	0.79–3.15	
Thioridazine	0.1	0.05	2.36	0.84–6.64	
Chlorpromazine^a^	0.1	0.02	6.31	1.34–29.69	
Sertindole	0.06	0.02	3.15	0.58–17.21	
Sulpiride	0.04	0	–		
Cyamemazine	0.01	0	–		
Pimozide	0.01	0	–		

^a^Statistically significant difference between patient groups (95% CI for RR)

During 2012, there were 7284 individuals with a diagnosis of schizophrenia, out of which 751 (10.3%) were dispensed long-acting injectables only and 914 (12.5%) both per oral and parenteral medication. In total, 1665 individuals were dispensed long-acting injectable antipsychotics (Table 3). Of the population with a NAP diagnosis, 2% were dispensed only parenteral antipsychotics and 3% per oral and parenteral antipsychotics.

Of those patients in the study population who had no health care consultations and received only pharmaceutical treatment in 2012, 90% were dispensed per oral antipsychotics, 5% long-acting injectable antipsychotics and 5% a combination of the two formulations of antipsychotics.

### Comorbidities

The most common comorbidities investigated were substance abuse/dependence and hypertension (Table 1).

### Concomitant drug treatment

**Fig. 2:**
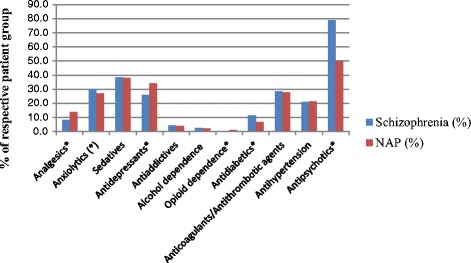
Dispensed prescriptions for concomitant pharmaceutical treatment in 2012 (% of respective patient group). *statistically significant difference between the diagnostic groups (95% CI for RR). Most medicines for treatment of nicotine addiction are available as over the counter medications. The number of individuals with dispensed prescriptions for this treatment is therefore small and in this case included in the anti-addictive category

We found statistically significant differences between the two psychosis populations in the concomitant drug treatment with opioids, anxiolytics, antidepressants, opioid dependence treatment, antidiabetics and antipsychotics. The schizophrenia patients were treated less often with antidepressants but more often with antipsychotics, antidiabetics and anxiolytics than the NAP population (Fig. 2).

## Discussion

In this study we found a prevalence rate of 3.5/1000 inhabitants for schizophrenia and 5.5/1000 inhabitants for NAPs. Antipsychotics were more commonly dispensed to the schizophrenia group than to the NAP group. Patients received medication per oral and as long-acting injectable medication, with long-acting injectables being more common in the patients with schizophrenia. Among the comorbidities we investigated, hypertension and drug dependence/abuse were most common in both patient groups, with a higher prevalence in the NAP group.

### Prevalence

Prevalence rates for schizophrenia usually range from 3 to 7/1000 [23]. Urban and migrant populations tend to show higher rates of illness [24–27]. The prevalence in our study is similar to the 3.7/1000 estimated in the Stockholm Non-Affective Psychoses Study (SNAPS) on a very similar population (82% coverage) in 2010 [27]. The SNAPS 1-year prevalence for all non-affective psychoses was 6.7/1000; we obtained a prevalence of 8.9/1000 when combining our patient groups in the same manner.

### Limitations of our analysis of disease prevalence

A diagnosis of schizophrenia is usually not registered in the medical records at first incident, but eventually patients not receiving a diagnosis of this chronic disease in primary care may be diagnosed in an outpatient specialist or inpatient setting. Hence, our estimates for the 1-year prevalence may be underestimated. Other potential causes for underestimation are avoidance of health care in general, poor illness insight (27–57% of patients with schizophrenia [5]) and poor adherence to treatment (41% of schizophrenia patients [14]).

### Health care use and need

Comparing our two patient groups for different levels of care, schizophrenic patients used psychiatric care more often. In addition, they had more outpatient consultations overall and more outpatient psychiatric consultations. The schizophrenia population was more likely to use only outpatient care.

Standard outpatient care for a schizophrenia patient includes regular check-ups for metabolic parameters, optimisation of medical treatment with antipsychotics and psychosocial measures. Consequently, the higher frequency of outpatient care (somatic and psychiatric) is not surprising. The reason for NAP patients using inpatient care more often is not known. However, we did see a higher prevalence of substance abuse and self-harm in the NAP patients, which might result in hospitalisations rather than outpatient visits.

### Antipsychotics: effect, dispensed prescriptions, route of administration and adherence

In our study population the antipsychotic substances dispensed mirrored prescription choices established in the national guidelines for medical treatment of schizophrenia. Olanzapine was dispensed at the highest rate to patients in both groups, with risperidone in third place in the schizophrenia group and second place in the NAP group. That olanzapine was used to such an extent despite its known metabolic side effects is probably because it is often initiated during the acute phase of treatment owing to its calming and sedative effects and its lack of acute extra-pyramidal side effects. Patients may have continued with this medication even during stabilisation and maintenance phases of treatment, where it would have been advantageous to switch to medications with little or no metabolic side effects. Zuclopenthixol, recommended for emergency treatment of patients with high levels of anxiety or aggressiveness, was second on the list of antipsychotics dispensed for the schizophrenia group and seventh in the NAP group.

For patients who received no dispensed prescriptions in 2012, there are methodological explanations (e.g., a patient’s death or migration out of the county, dispensation of drug outside the time frame of the study or drugs dispensed during hospitalisation) and treatment-related explanations (e.g., no antipsychotics were prescribed or non-adherence to treatment).

Adherence to treatment might be influenced by lack of illness insight, drug or alcohol abuse, side effects of medication and the route of administration. Administration by the parenteral route is usually not the first choice because it has a ‘lock-in’ effect on the chosen substance and dose over a longer period as compared with the per-oral route. A ‘trial and error’ approach is often necessary to determine the optimal treatment, and not all substances are available in both formulations. The use of per oral and parenteral routes of medications in the same year for a patient may reflect this approach, as well as the need for a ‘run-in phase’ during which the patient’s tolerability of the actual medication and the appropriate dosage are investigated.

### Comorbidity and concomitant drug treatment

It is not clear to what extent schizophrenia itself might contribute to the high prevalence of some of the medical comorbidities seen in patients with schizophrenia, or if these occur as side effects of antipsychotic medication [20]. Comparing comorbidities in the patients with schizophrenia with those of the patients with NAP might help elucidate some of these questions. Numerous studies have shown that patients with schizophrenia have a shorter life expectancy compared with a healthy population [28]. In the present study we analysed somatic co-morbidities associated with metabolic syndrome. Surprisingly, the 1-year non-adjusted prevalence for obesity in our study was only 2.2% for both diagnostic groups. This per cent is low compared with the results from the general population survey of Stockholm County in 2010, where Andersson et al. observed an age-standardised prevalence of 11.2% for men and 10.3% for women [29]. Obesity in patients with schizophrenia is perhaps not registered as a diagnosis per se, but can be regarded as an effect of antipsychotic medication or rooted in a particular aspect of the patients’ general behaviour.

In our study population we observed a statistically significant difference in the 1-year diabetes prevalence rate: 6.9% in schizophrenia patients and 4.8% in NAP patients. In comparison, the Finnish Health 2000 general population study showed an age- and sex-adjusted prevalence of 22% of type 2 diabetes in schizophrenic patients and 6% in a mentally healthy population [18]. Our lower prevalence estimate in the schizophrenic patients could be an effect of under diagnosis of schizophrenic patients’ somatic illnesses and to non-adjustment. In the NAP patients the prevalence estimate of 4.8% is somewhat lower than the results from a cross-sectional survey on the Swedish general population from 2009, which estimated a non-adjusted 1-year diabetes prevalence of 5.8% in women and 6.5% in men [30]. However, our result is similar to the Stockholm County population survey of 2010 in which Andersson et al. demonstrated an overall age-standardised 1-year prevalence of 4.6% [29].

We have investigated dispensed prescriptions in 2012 for the chosen diagnoses co-occurring with schizophrenia. In agreement with the diagnostic statistics the number of patients with dispensed prescriptions for antidiabetics was statistically significantly higher in the schizophrenic population than in the NAP population.

Our observed substance abuse/dependence 1-year prevalence was 7.9% in the schizophrenia population and 11.7% in the NAP population of Stockholm County. Calculated over a longer period (2000–2012), we observed a prevalence of 24.8% in the schizophrenic patients and 28.4% in the NAP patients. The excess of substance abuse in the NAP population was statistically significant for both the 1-year and 12-year prevalence rates.

We found a statistically significant difference in self-harm in our study material, with a higher rate in the NAP population (1-year prevalence of 0.9 vs. 0.6%). This finding may reflect the more regular visits with a physician in the schizophrenia population, which would include monitoring the risk of suicide and self-harm.

### Strengths and limitations

Swedish health registries are available on a national and regional level. These data are of high quality, and because they are based on a personal identifier common to all national registries, can easily be linked through record linkage. In a validation study the National Patient Registry (maintained by the Swedish National Board of Health and Welfare) has been shown to have high validity [31]. Because VAL is used as provider of data for updating the National Patient Registry, this is an indication of the accuracy and validity of our source. In primary care, though, the registration of diagnoses is not as thorough as that in the in- and outpatient registries. Data on private health care is not available but a study in early 2000 showed that ˂10% of the psychiatric patients in Stockholm had private psychiatric health care. For those patients who only consulted private health care, their data on health care consumption might be missing [32]. However, their dispensed prescriptions are paid for by the Stockholm County council and therefore these patients are most likely part of our study population.

Although the Stockholm population overall is younger and has a higher educational level and mean income than the rest of the country, the region is very diverse in such domains as geography, housing and social settings. An additional strength of this study is that we have a total coverage of the entire region and have separated the patients with schizophrenia from those suffering from other types of NAP in our analyses.

Because not all medications are dispensed but to some extent administrated in hospital settings, the interpretation of the amount and distribution of dispensed medications is compromised. The parenteral route of administering medications was more often conducted in a hospital setting than the per oral medications. Approximately 30% of the parenteral route of administration and some 10% of the per oral medications are administered in a hospital setting and not registered on an individual basis (personal communication). This circumstance leads to an underestimation of the rate of consumption of antipsychotics in general and for the parenteral form in particular in our study.

Finally, we observed a surprisingly high rate of NAPs in the oldest population. We also found prevalence rates of comorbidities not corresponding to the expected prevalence rates of treatment for these comorbidities. These findings could indicate inappropriate treatment, but also quality problems in the recording of diagnoses as well as potential progression from NAP to schizophrenia. The cross-sectional study design did not allow further analysis of this issue. Thus, longitudinal studies on the pharmacoepidemiology, comorbidity (diagnosis and concomitant medical treatment) and socio-demographic factors of schizophrenia are suggested.

## Conclusions

By analysing the two mutually exclusive patient groups separately with a cross-sectional design, we could observe differences between the groups in almost every aspect investigated. To thoroughly investigate the causes of these differences and the potential progression from a diagnosis of NAP to one of schizophrenia a longitudinal study design is recommended.

## Abbreviations

CATIE: Clinical Antipsychotic Trials of Intervention Effectiveness
ECA: Epidemiological Catchment Area study
ICD: International Classification of Diseases
NAP: Other non-affective psychosis; ICD-10 F21-F29
VAL: Database of health care provided in Stockholm County which is financed by tax money
